# Highly Specific Detection of Oxytocin in Saliva

**DOI:** 10.3390/ijms24054832

**Published:** 2023-03-02

**Authors:** Muhit Rana, Nimet Yildirim, Nancy E. Ward, Stephanie P. Vega, Michael J. Heffernan, Avni A. Argun

**Affiliations:** 1 Giner, Inc., 89 Rumford Avenue, Newton, MA 02466, USA; 2 Raptamer Discovery Group, 3900 Essex Lane, Suite 575, Houston, TX 77027, USA

**Keywords:** oxytocin, aptamer, peptide detection, electrochemical, sensor, saliva

## Abstract

Oxytocin is a peptide neurophysin hormone made up of nine amino acids and is used in induction of one in four births worldwide (more than 13 percent in the United States). Herein, we have developed an antibody alternative aptamer-based electrochemical assay for real-time and point-of-care detection of oxytocin in non-invasive saliva samples. This assay approach is rapid, highly sensitive, specific, and cost-effective. Our aptamer-based electrochemical assay can detect as little as 1 pg/mL of oxytocin in less than 2 min in commercially available pooled saliva samples. Additionally, we did not observe any false positive or false negative signals. This electrochemical assay has the potential to be utilized as a point-of-care monitor for rapid and real-time oxytocin detection in various biological samples such as saliva, blood, and hair extracts.

## 1. Introduction

Oxytocin (OT) is a neuropeptide hormone best known for its role in the facilitation of childbirth through the induction of myometrial smooth muscle contractions [1,2]. It also plays an essential role in the later stages of life, affecting various health conditions and complex social behaviors. These include affiliation, sexual behavior, social recognition, social bonding, parturition, lactation, appetite regulation, aggression, depression, obesity, and social deficit of autism spectrum disorder (ASD) [3,4,5,6,7]. Recent scientific evidence indicates that dysfunction of the oxytocin system could be the underlying cause for the pathogenesis of insulin resistance and dyslipidemia and contribute to weight gain in some genetic obesity conditions such as the Prader–Willi syndrome (PWS). The circulating peripheral oxytocin levels were reported to be higher in children with PWS as compared to their healthy siblings; in contrast, the oxytocin levels were lower in individuals with anorexia [5,8,9,10,11,12,13,14,15]. Oxytocin is also involved in regulation of metabolic energy and linked to late-onset obesity in an oxytocin receptor-deficient mice model [12]. Therefore, monitoring oxytocin levels could play a therapeutic role in management of obesity and diabetes. Besides, it has been suggested that oxytocin, known to promote mother-infant bonds, may be implicated in the social deficit of autism [6]. The researchers recently reported that oxytocin levels were significantly lower in individuals with ASD as compared to control subjects [16,17,18]. Some features of ASD have also been linked to disturbance of the oxytocin system in the body [19,20,21]. Exogenous administration of oxytocin has improved various outcomes associated with social responsiveness, including eye contact, emotion recognition, social cognition, and neural circuitry associated with social awareness [17,18,22].

OT is of particular interest in the study of childbearing women, as it has a role in the onset and course of labor and breastfeeding. One in four births worldwide (more than 13 percent in the United States) is induced with oxytocin [23]. Exogenous administration of oxytocin is critical in a clinical setting for induction and augmentation of labor as well as management of postpartum uterine atony/hemorrhage [24,25,26,27,28]. When oxytocin levels are high, strong contractions occur that reduce the chance of bleeding or postpartum hemorrhage. A double-blinded clinical trial on 200 participants showed oxytocin’s role in reducing blood loss during cesarean delivery and the investigators reported that oxytocin infusion is an appropriate regimen [29,30]. Another clinical study by the Cohen Group showed that obese patients required more oxytocin than lean women during the first stage of successful labor induction, indicating that the current clinical practice can benefit from dosage optimization [31]. 

Oxytocin levels also have great significance during the perinatal period. For example, endogenous oxytocin is a potential biomarker for the prediction of the type of labor and risk assessment of premature labor. Perinatal screening after the 32nd week of pregnancy can help predict premature labor in high-risk pregnancies [32]. Increased levels of circulating peripheral oxytocin levels are linked to postpartum breastmilk production as well as a decrease in the frequency of migraine headaches over the course of pregnancy [33,34,35]. Additionally, a group of physicians and researchers from Northwestern and Indiana University conducted multi-centered clinical research on 66 pregnant women and reported that elevated oxytocin levels during pregnancy may signal postpartum depression (PPD) [36]. 

Oxytocin also plays a critical role as a neurotransmitter. It mediates the brain’s dynamic function and various complex social behaviors, including affiliation, sexual behavior, social recognition, and aggression. Oxytocin is secreted in the hypothalamus along with a similarly structured nonapeptide called vasopressin. Due to the similar structure of these two neuropeptides, they often compete to bind with existing antibodies, resulting in poor specificity for current immunoassays. Currently, two existing immunoassays (radioimmunoassay-RIA and enzyme immunoassay-EIA) are insufficient for sensitive and specific detection of oxytocin. This problem can be resolved with mass spectrometry combined with liquid chromatography (LC/MS); however, micro dialysis of the sample and lengthy retention times make this method unsuitable for practical oxytocin monitoring.

Despite being one of the most widely utilized drugs in obstetrics, there is currently no instrument capable of point-of-care (POC) detection of oxytocin. While its pharmacokinetics has been extensively studied for both intravascular (IV) and intranasal (IN) administration, its dose-effect response has been poorly understood. The research studies, as well as the clinical significance of perinatal oxytocin, suggest that accurate and real-time measurement of peripheral oxytocin levels may help develop pharmacokinetic models to facilitate a better understanding of the effects of oxytocin and optimize oxytocin use. [37,38] Therefore, it would be extremely valuable for researchers and medical professionals to have a simple and practical assay that would accurately determine the peripheral levels of oxytocin in pregnant women and guide clinical plans for oxytocin administration. Clinical researchers could also benefit from the development of such a tool to aid in quantifying peripheral oxytocin levels toward a better understanding of the long-term effects of exogenous oxytocin on mother and child. 

The biological levels of oxytocin in bodily fluids such as saliva are very low. The physiological level of peripheral oxytocin is only on the order of 1–300 pg/mL, making it difficult to detect it with high specificity using the current immunoassay-based methods. These antibody-based methods also suffer from significant cross-reactivity by arginine vasopressin, another similar neuropeptide hormone [39,40,41]. Other laboratory-based methods, such as LC-MS/MS, exist but they require complex instrumentation and sample processing steps, increasing the cost and turnaround times. 

**Figure 1:**
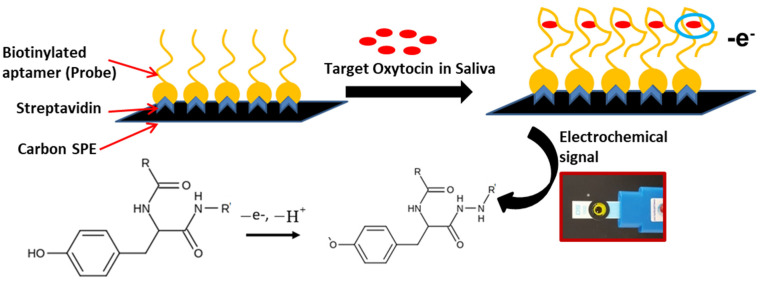
Electrochemical oxytocin detection scheme on aptamer-modified carbon electrode (a-SPE) surface. The proposed oxidation mechanism of oxytocin at the tyrosine moiety. R and R′ represent the remaining amino acid parts of the oxytocin.

As illustrated in Figure 1, we have developed an aptamer-based electrochemical assay that enables the measurement of oxytocin in minimally invasive biological samples (e.g., commercially available pooled saliva samples) with high sensitivity and specificity while lowering the detection limits to pg/mL levels. Upon running a rapid (<2 min) electrochemical algorithm, the oxytocin content is quantified. This study demonstrates the electrochemical oxytocin detection in both lab samples and exogenously enriched saliva samples with a limit of detection (LOD) of 1 pg/mL. Table 1 shows the comparative performance of our electrochemical sensor assay with other available technologies, including the commercially available ELISA kit.

## 2. Results

### 2.1. Aptamer Development 

Aptamers are synthetic, single-stranded DNA or RNA oligonucleotides with very high affinity, selectivity, and specificity to low molecular weight molecules, macromolecules such as proteins, and even whole cells [46]. Aptamers have been generated with binding constants (K_d_) to their targets that are in the nanomolar range, comparable to antibody-antigen values. Commonly used bioreceptors (enzymes and antibodies) are mostly unavailable for small peptide targets, especially for short-chain peptides, making aptamers excellent candidates for bimolecular recognition due to the small size of nucleic acids and their versatile in vitro development and synthesis for any targeted peptide. In comparison to antibodies and enzymes, aptamers are also less prone to degradation and denaturation. 

Aptamer development has traditionally been via an iterative process called systematic evolution of ligands by exponential enrichment (SELEX); however, this approach has limited aptamer development studies for new targets to academic laboratories or specialized companies. The emergence of oxytocin aptamer as “Raptamers” from Raptamer Discovery Group has been a game changer in this field to allow efficient aptamer development for a wide range of targets. We have employed this non-SELEX strategy in our study to develop the first aptamer specific to the oxytocin molecule. In contrast to SELEX, the Raptamer strategy employs a bead-based library as the basis for the rapid selection of affinity agents for targeted biomarkers with standard laboratory practices. The Raptamer selection process has the advantage of using a single round of PCR amplification; this is in contrast to the multiple rounds of PCR in SELEX which can lead to PCR bias in the aptamer selection. In addition, Raptamer library beads incorporate proprietary modified nucleotides in the random region; these modified bases provide a more functionally diverse composition for enhancement of interactions with target molecules. In our study to develop an oxytocin aptamer, the combinatorial library (typically ~10 × 10^−7^ members) was initially mixed with magnetic particles functionalized (tagged) with oxytocin molecule. Isolation from magnetic separation provided the first stage selection of library beads, and the putative Raptamers were released and subjected to a secondary pull-down to remove ‘false-positive’ candidates. The true Raptamers were identified using next-generation sequencing (NGS) methods and the comparison of the amount of each sequence after pull-down to its initial presence in the solution pool. The most abundant sequences were then synthesized with the appropriate oligonucleotide modifications and end modifications such as biotinylation.

### 2.2. Selection of Aptamers for Oxytocin

**Figure A1:**
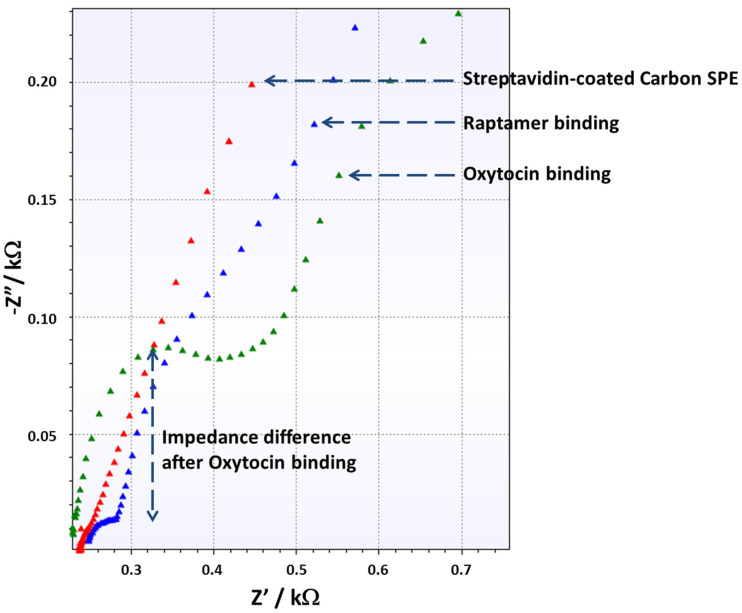
Nyquist plots for Streptavidin coated Carbon SPE, biotinylated Raptamer immobilization onto the electrode and Oxytocin binding to the Raptamer.

**Figure A2:**
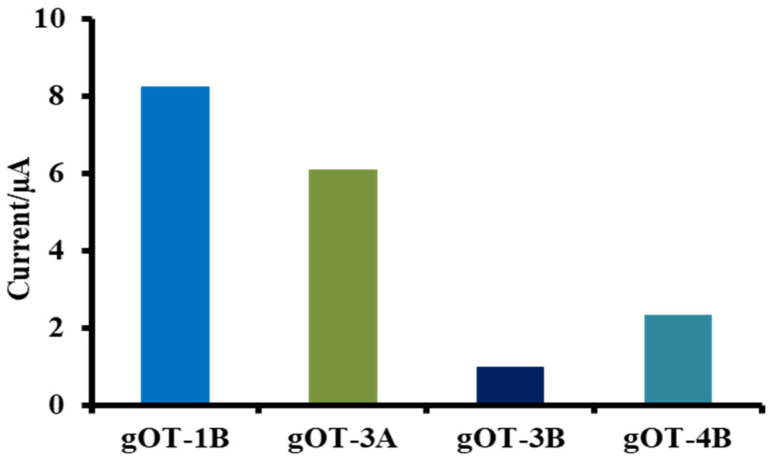
Electrochemical sensor signal response to oxytocin from four candidate aptamers is shown as a bar chart, verifying the strong affinity of gOT-1B.

After the initial bead assay and the NGS stage, eight putative Raptamer sequences for oxytocin were obtained. All of the eight putative Raptamers were biotinylated, immobilized on streptavidin-coated carbon screen-printed electrodes (SPE), and characterized for oxytocin binding using both electrochemical impedance and direct electrochemical oxidation as demonstrated in Figure 1. Oxytocin was initially introduced to the Raptamer-modified SPEs (a-SPE) in controlled buffer solutions and incubated for durations varying between 1 and 10 min. After a brief rinsing step, aptamer-bound oxytocin resulted in impedance changes on the surface of the electrode (Figure A1) and the magnitude of this change was used to rank the affinity of each Raptamer as shown in Table 2 and Figure A2. This initial Raptamer validation step allowed us to rapidly down select four Raptamers for further characterization using an electrochemical oxidation method (utilizing the electrochemically active tyrosine group in oxytocin) and an optical particle aggregation method (using wavelengths shifts of functionalized gold nanoparticles).

All the measurements were recorded as Nyquist plots in a 0.1 M PBS buffer solution containing 5 mM [Fe(CN)6]^3/4^ redox pair (1:1 M ratio). The electrochemical impedance spectroscopy (EIS) spectra were conducted over a frequency range from 10 kHz to 0.1 Hz using an AC voltage with amplitude of 10 mV, superimposed on a DC potential of 0.15 V vs. Ag/AgCl. Affinity levels of each oxytocin aptamer (Raptamer) to oxytocin is denoted as “-” for no affinity, “+” for weak affinity and “++” for strong affinity as shown in Table 2.

### 2.3. Validation of Aptamers via Spectroscopic Characterization 

**Figure 2:**
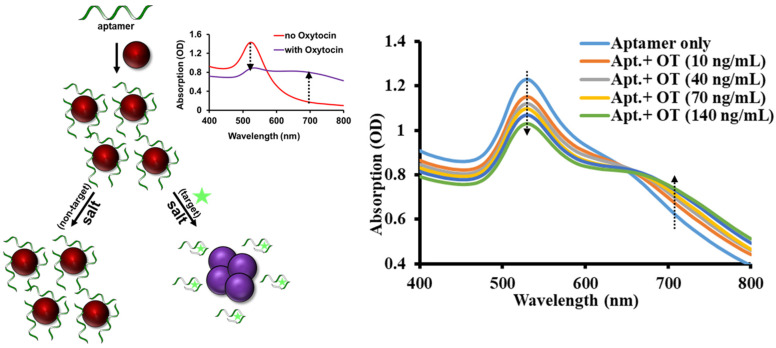
Gold nanoparticle assay scheme. Raptamer with a random coil structure binds to gold nanoparticles (AuNP). Binding induces a conformation change to a rigid stem-loop structure and the AuNPs aggregate, leading to absorption changes at 520 nm and 700 nm. The green star represents oxytocin targets.

We performed an independent validation of one of the candidates Raptamers, and then we have developed a robust, non-electrochemical procedure in house to validate the performance of this Raptamer. We utilized a well-established gold nanoparticle colorimetric assay, which proved to be an independent confirmation of aptamer binding. Our detection strategy [47] along with the spectroscopic information to characterize and validate each Raptamer is demonstrated in Figure 2. Briefly, citrate-reduced gold nanoparticles (AuNP) possess negative charges and their strong inter-particle electrostatic repulsive forces make them retain a characteristic red color in the solution. Upon mixing, the aptamer adsorbs on negatively charged AuNP and protects the nanoparticle against positively charged salt (Na^+^)-induced aggregation with its negative phosphate backbone. Conversely, when target biomarker (oxytocin) is introduced, the adsorbed aptamer desorbs from AuNP surface and strongly binds to the target, leaving AuNP unprotected in the solution. In presence of ~150 mM NaCl, the remaining AuNP negative charge is easily neutralized with Na^+^, leading to a loss in electrostatic repulsion. As a result, the inter-particles distance reduces and a salt-induced aggregation takes place and leads to a plasmon effect reflecting in color transition (red to purple to clear) in less than a minute [48,49,50]. In fact, the gold nanoparticles surface plasmon resonance peak (OD_520_) is reduced and the peak is shifted to a longer wavelength region (OD_700_) with increasing amount of Na^+^ ions. This simple mechanism allowed us to discriminate aptamer functionality in complex biological matrices like saliva based on the quantitative information obtained using UV-Vis spectroscopy for cross-validation. 

**Figure A3:**
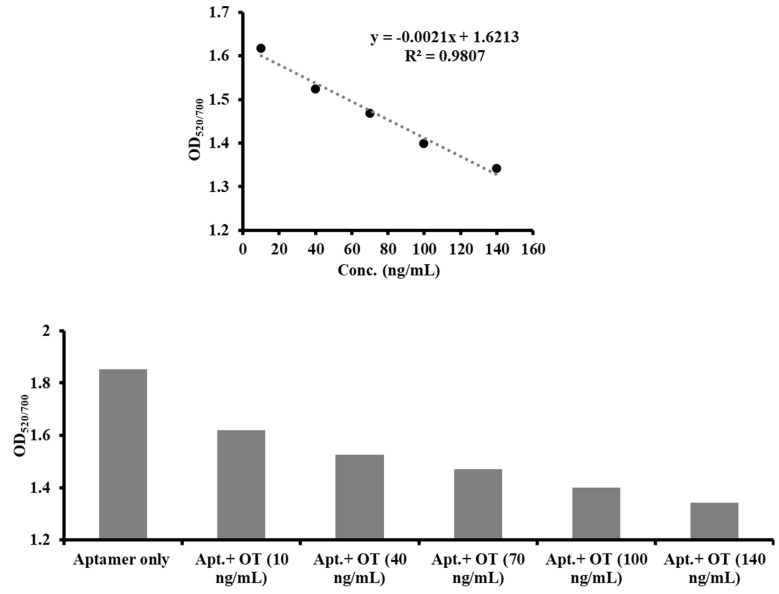
Sensitivity analysis of the colorimetric assay. **Top:** Linear regression produced a calibration curve with an R^2^ value of 0.9807. **Bottom:** Dose-dependent results were observed from the AuNP assay in the buffer.

With the colorimetric assay, we performed sensitivity and specificity analysis of Raptamer gOT-1B. Figure 2 and Figure A3 with inset demonstrates a dose-dependent linear correlation between the absorbance reading (OD_520_) and various oxytocin levels. As expected, a more drastic color change was observed when higher dosing of OT was introduced in buffer. According to the absorbance reading at 520 nm wavelength, a calibration curve was obtained with a coefficient of determination (R^2^ value) of over 0.98 after linear regression. These findings prove that Raptamer gOT-1B (one of the putative aptamers) is capable of distinguishing different levels of oxytocin target. 

**Figure 3:**
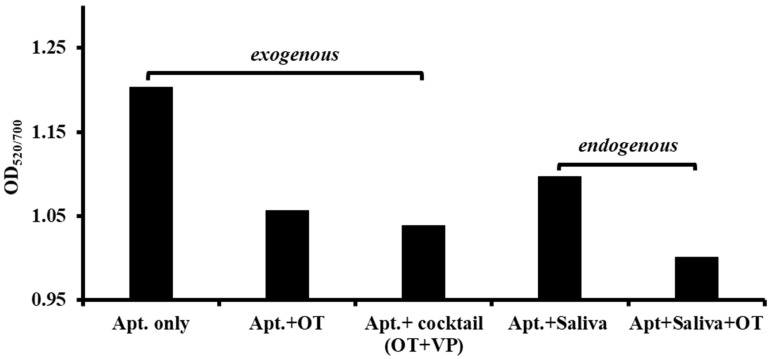
Exogenous and endogenously expressed oxytocin detection and their specificity analysis. Raptamer conc. was 400 nM (ng/mL), OT = 100 ng/mL, VP = 140 ng/mL.

In this gold nanoparticle aptamer assay, when oxytocin was exogenously exposed to Raptamer-bound nanoparticles, a signal reduction at OD_520_ confirmed the binding event as shown in Figure 3. When a cocktail of oxytocin and vasopressin was tested exogenously, the absorbance reading showed nearly no difference compared to the result when only oxytocin was present. It is crucial to distinguish oxytocin from vasopressin since the two molecules differ only by two peptide residues while both contain the signal-generating tyrosine in their structures [1]. In this case, the absence of any false positive or false negative detection verified the specificity of the chosen aptamer. The specificity demonstrated by our novel technology offers a distinct advantage over commercially available immunoassays such as EIA or RIA [41]. A reduction in absorption was also observed when additional oxytocin was added endogenously, confirming the proper function of aptamer in real saliva environment. At this point, we have successfully validated the functionality of the Raptamer candidate gOT-1B identified previously for oxytocin detection both exogenously in buffer and endogenously in saliva.

### 2.4. Electrochemical Detection of Oxytocin

**Figure 4:**
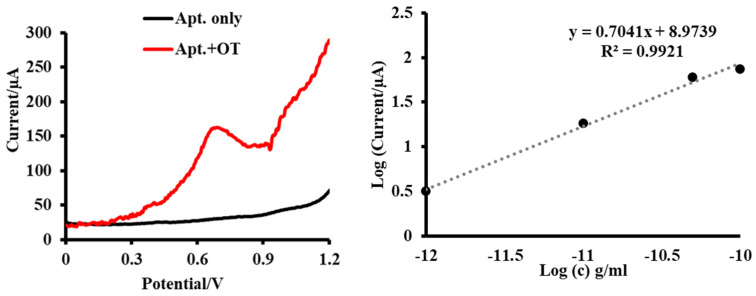
Sensitivity analysis. (**Left**) Raptamer gOT-1B binding to oxytocin confirmed via the electrochemical signal. (**Right**) Calibration curve. Experiments were performed oxytocin concentrations of 1 pg/mL, 10 pg/mL, 50 pg/mL, and 100 pg/mL.

With the selected and validated Raptamer, we continued to develop an electrochemical assay for oxytocin detection. As highlighted in Figure 4, oxytocin aptamer gOT-1bB produced sufficient and distinguishable signals when binding with exogenously expressed oxytocin. The sensitivity analysis showed a dose-dependent response curve as demonstrated in Figure 4 (right panel). We obtained a calibration curve to correlate the electrochemical signal with oxytocin concentrations in reaction buffer environment.

**Figure 5:**
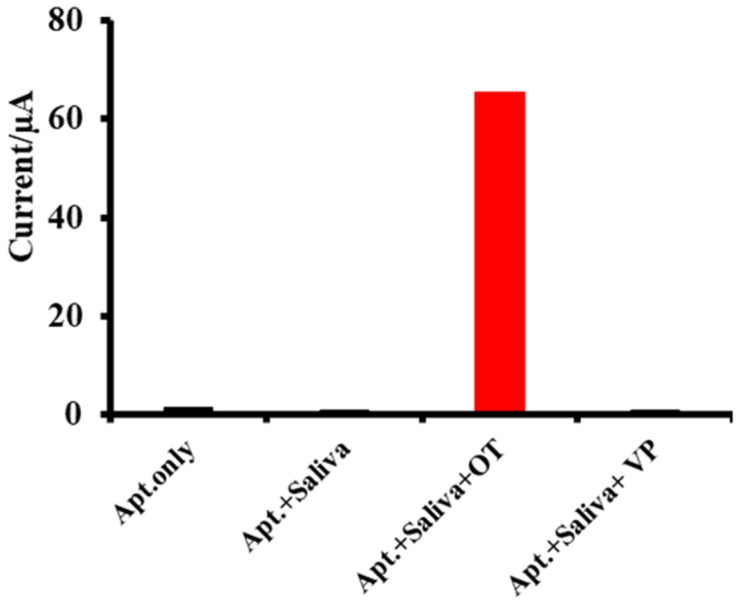
Specificity analysis. 1 µg/mL oxytocin and >100 µg/mL vasopressin (VP) were spiked separately in apparently healthy (control) saliva samples.

As Figure 5 shows, when oxytocin was exogenously administrated in saliva, the aptamer on the carbon SPE surface bound specifically to oxytocin, and the tyrosine residue of the peptide produced a peak current as signal readout as shown in Figure 4. Such peak current (readout signal) was only observed when exogenously oxytocin was introduced into test samples both in buffer and control saliva environment [51,52]. On the other hand, adding vasopressin to saliva did not produce any signals, confirming that oxytocin was the only target.

## 3. Discussion

In this study, we successfully established an electrochemical sensor and a technology platform for future development of a rapid and accurate instrument capable of measuring oxytocin level in peripheral body fluids at point-of-care. This novel technology utilizes Raptamer-modified disposable carbon electrodes to achieve preeminent sensitivity of 1 pg/mL, which is on the order of laboratory-based technologies such as LC-MS and more sensitive than the commercially available Enzo Oxytocin ELISA Kit [41].

Cross-validated by electrochemical impedance spectroscopy and a nanoparticle colorimetric assay, we confirmed that this electrochemical detection method is also highly specific to oxytocin (1 µg/mL) with 100× specificity over vasopressin (>100 µg/mL) as shown in Figure 5. While the current commercially available immunoassays such as RIA and EIA often fail to distinguish vasopressin and oxytocin, our approach is able to capture the structural difference of these two similar molecules.

## 4. Materials and Methods

### 4.1. Materials and Equipment

Invitrogen’s Ultrapure DNase-free, RNase-free DEPC treated water (catalog # 4387937) was used in all studies. 10X PBS, NaCl, MgCl_2,_ and all other reagents were purchased from Sigma-Aldrich, St. Louis, MO 63103, USA. Oxytocin peptide (ab120186) is purchased from Abcam Inc. Cambridge, MA 02139, USA. To avoid any DNase contamination, DNA Away (DNA Surface Decontaminant) was purchased from Thermo Scientific and used before performing any experiment. Streptavidin screen-printed carbon electrodes (Catalog# Dropsens DRP-STR110) were purchased from Metrohm USA Inc., Riverview, FL, USA. The USB-powered potentiostat (Model number: EmStat3+, Potential range ±3 V or ±4 V, and current ranges 1 nA to 10 mA or 100 mA) was obtained from PalmSens BV, Houten, Netherlands. A Raptamer (formerly X-Aptamer) Selection Kit was purchased from Raptamer Discovery Group, Houston, TX (RDG; formerly AM Biotechnologies; Houston, TX). This kit employs a proprietary bead-based library, containing modified DNA nucleotides within the random region, as the basis for the rapid selection of affinity agents for several targets in parallel using standard laboratory tools. All commercial de-identified pooled saliva samples were obtained from BioIVT (Westbury, NY, USA) and tested at a BSL-2 lab facility.

### 4.2. Raptamer Selection

For the primary Raptamer selection, a non-SELEX bead-based selection approach was utilized. In this selection, a bead-based DNA oligonucleotide library was mixed with oxytocin-coated magnetic particles and incubated for 90 min at room temperature. The library beads containing oligonucleotides that bound to oxytocin were isolated via magnetic separation. The isolated library beads were resuspended in 1N NaOH and incubated at 65 °C for 30 min to cleave the oligonucleotides from the beads. The cleaved oligonucleotides were then subjected to a secondary pull-down selection to remove ‘false-positive’ binders and to enrich the pool for Raptamers with high affinity to oxytocin. Following PCR amplification of the enriched and control pools and next-generation sequencing (PrimBio Research Institute, Garnet Valley, PA, USA), the candidate oxytocin Raptamers were identified by a proprietary analysis method (Raptamer Discovery Group, LLC, Houston, TX, USA). This method identifies the sequences enriched in the primary target pool compared to the control(s), which consist of any negative target controls and the magnetic particle (not containing target) control. These enriched oxytocin candidate Raptamers were then synthesized with the appropriate modified nucleotides included in the sequences.

### 4.3. Gold Nanoparticles (AuNPs) Synthesis for Nanoplasmonic Assay

Gold nanoparticles (AuNPs) were synthesized using the standard citrate reduction method. This nano-plasmonic test was designed according to the published articles [49,53,54,55]. Briefly, 2 mL of 50 mM HAuCl_4_ was added into 98 mL of boiling DI water in an Erlenmeyer flask. Then 10 mL of 38.8 mM sodium citrate was added, and the mixture was stirred until the color turned wine-red. The synthesized homogenous gold nanoparticles were characterized using UV-Vis spectroscopy and stored at 4 °C. All aptamers were reconstituted in 1× PBS, 2 mM MgCl_2_, pH 7.4, and targets were resuspended in 1 × PBS. All oxytocin aptamers were pre-heated at 95 °C for 5 min to remove any dimerization before utilizing in any experiment.

### 4.4. Nanoplasmonic Aptamer Characterization and Identification

For aptamer validation, 1 µL of 10 µM aptamer is added to 98 μL of 11 nM AuNPs (~13 nm size) to a final volume of 99 µL and incubated at room temperature (RT) for 5 min. After 5 min, 1 µL of 10 µM target (OT) is added to the 99 μL of pre-incubated Aptamer/AuNP solution for a final volume of 100 μL, resulting in final aptamer and target concentrations of 100 nM. After an additional 15–20 min of incubation at RT, 3 µL of 1 M of NaCl is added to 100 µL of the nanoparticle solution to a final concentration of ~30 mM Na^+^. After the addition of NaCl, the color transition was observed within 1 min or less and recorded with a photograph. In the presence of salt addition, when the aptamer binds to the specific target, it desorbs from the gold nanoparticle surface, leaving gold nanoparticles unprotected and easily neutralized by Na^+^ and showing a color changed from red to purple. Similarly, if the aptamer does not bind to its target, the gold nanoparticle’s color will be unchanged upon salt (Na^+^) addition. The resulting nanoparticle assembly’s change in the optical density (OD) at 520/700 nm (Abs 520/700) was used to plot the aggregation rate and degree. The UV-Vis spectrum of each sample was measured in 96 well plates using BioTek microplate reader (Gen5 microplate data collection and analysis software, BioTek Science Company, Winooski, VT). Control experiments were performed in the absence of target (only Aptamer + AuNP + adjusted reaction buffer, 1 × PBS, 2 mM mgCl_2_, pH 7.4). We utilized a similar procedure for detecting and validating all eight-oxytocin candidate Raptamers in buffer and saliva samples. In the previous study, the change in OD ratio at 520/700 nm of the resulting nanoprobe complex assembly was used to determine the limit of detection (3 σ/slope) where σ is the standard deviation of controls while slope is obtained by linearly fitting the calibration curve [53,54]. The limit of detection (LOD) is calculated, followed by the 3-sigma rule. The equation is, LOD =3.3 × standard deviation of the regression line (σ) /Slope(S). A 3σ-rule is widely used to determine the signal-to-noise ratio for estimating the detection limit [54,55,56,57,58,59].

### 4.5. Sensitivity Measurements

For sensitivity measurement, studies were also performed using various amounts (0, 10, 40, 70, 100, 140 ng/mL) of target OT in 100 μL of solution and, color changes were recorded with in 1 min after incubation with ~30 mM NaCl. Control experiments were performed in the absence of target OT. We performed similar procedure to detect oxytocin in Saliva where we spiked various amounts of target in presence of fixed aptamer concentration. The OD value at 520/700 nm and the pictures of the nanoparticle suspensions were recorded. All experiments were performed in triplicate (*n* = 3) using 96 well plates. For specificity measurements, individual OT Raptamers with their target and/or non-target analyte with a ratio of Raptamer: target = 1.4:1 (for buffer/Saliva) and evaluated to verify its false positive and false negative binding performance. The change in OD value at 520/700 nm was measured and plotted.

### 4.6. Electrochemical Measurements (Sensitivity, Specificity and Cross-Reactivity)

For electrochemical sensitivity measurement, various concentrations (1 pg/mL to 100 pg/mL) of target oxytocin were tested, and a dose-dependent calibration curve with improved sensitivity (R^2^ = 0.9921) is observed from saliva samples. The assay’s specificity and cross-reactivity is evaluated in presence of non-target (Vasopressin) with saliva samples. A complex cocktail mixture of non-target peptide was prepared to show that the assay can detect only the targeted oxytocin in vitro PBS buffer as well as in exogenously enriched commercial saliva samples.

### 4.7. Electrochemical Detection of Oxytocin in Spiked Saliva Sample

The unprocessed (no anticoagulant/filtration, storage condition at −20 °C or colder) human saliva samples (de-identified, pooled samples sourced from BioIVT) were utilized to demonstrate the assay preclinical utility. Prior testing, 70 µL of 400 nM oxytocin aptamer in 1× PBS 2 mM MgCl_2_ is loaded on s-SPEs (prior washed with 1× PBS) for at least 10 min, then 70 µL (35 µL saliva + 35 µL of 1× PBS, 2 mM MgCl_2_) loaded on oxytocin aptamer immobilized streptavidin (s)-SPEs. Detection time is less than 5 s. The electrochemical Square Wave Voltammetry (SWV) parameters are: Current: 1 mA; T equilibration: 0 s; E begin: 0.0 V; E end: 1.5 V; E step: 0.005 V; Amplitude: 0.05 V; Frequency: 15.0 Hz.

## Figures and Tables

**Table 1:** Summary of Baseline Oxytocin measurement in various studies in human plasma and saliva.

Subjects	Oxytocin (pg/mL)	Extracted	Assay	Source	Ref.
Healthy Adults	~2.0	Yes	RIA	Saliva	[42]
Healthy women	1.66	Yes	RIA	Plasma	[43]
Healthy Men	92.3	No	EIA	Plasma	[44]
Healthy Adults	2.58	Yes	LC-MS	Plasma	[45]
Commercial Kit (Enzo Kit)	15	N/A	ELISA	Body fluids	[41]
Commercial Pooled Saliva Samples (BioIVT)	1.0	No	Electrochemical	Pooled Saliva Samples	This work

**Table 2:** Exemplar affinity results from impedance measurements. Affinity levels of each oxytocin aptamer (Raptamer) to oxytocin is denoted as “-” for no affinity, “+” for weak affinity and “++” for strong affinity.

Giner’s Oxytocin (gOT) Aptamers	Affinity Level for gOT
gOT-1A	**-**
**gOT-1B**	**++**
gOT-2A	**-**
gOT-2B	**-**
**gOT-3A**	**++**
**gOT-3B**	**+**
gOT-4A	**-**
**gOT-4B**	**++**

## Data Availability

Data available on request.

## Appendix A

Upon selection of four Raptamers using the impedance study (Figure A1), electrochemical oxidation of Raptamer-bound oxytocin was carried out to select the best Raptamer suitable to develop an electrochemical assay. As highlighted in Figure A2, all four Raptamers produced sufficient and distinguishable signals when binding with exogenously expressed oxytocin. Among the four, gOT-1B showed the most sensitive response with the strongest signal.
